# Editorial: Determinants and Impact of Early Vascular Aging in Children and Adolescents

**DOI:** 10.3389/fped.2022.871524

**Published:** 2022-03-30

**Authors:** Ruan Kruger, Mieczyslaw Litwin, Rachel E. Climie

**Affiliations:** ^1^Hypertension in Africa Research Team (HART), North-West University, Potchefstroom, South Africa; ^2^MRC Research Unit for Hypertension and Cardiovascular Disease, North-West University, Potchefstroom, South Africa; ^3^Children's Memorial Health Institute (IPCZD), Warsaw, Poland; ^4^Menzies Institute for Medical Research, University of Tasmania, Hobart, TAS, Australia; ^5^Baker Heart and Diabetes Institute, Melbourne, VIC, Australia; ^6^Université de Paris, INSERM, U970, Paris Cardiovascular Research Center (PARCC), Paris, France

**Keywords:** adolescents, arterial stiffness, blood pressure, cardiovascular health, children, early vascular aging, preventative cardiology

Cardiovascular health is essential for longevity and limited morbidity with increasing age. However, exposure to several risk factors from as early as preconception (genetic or epigenetic predispositions, lifestyle and behavioral risk factors, and early life programming) may probe accelerated vascular deterioration, also known as early vascular aging (EVA). The central hallmark of EVA is arterial stiffness, although several other markers have been proposed to identify individuals at increasing risk of accelerated biological aging. The collection of articles published under this Research Topic addresses the need for more scientific evidence to refine the current concepts of EVA within the scope of pediatrics.

Early life development or early life programming is a critical period in which a fetus' biological trajectory is determined for cardiovascular disease susceptibility. In a prospective birth cohort study by Wang et al., maternal gestational weight gain, as one of the early life programming risk factors, was measured before pregnancy, in the first trimester (≤ 12 weeks), and before delivery. The aim of their study was to determine whether excessive maternal gestational weight gain, as an adverse intrauterine environment, could contribute to alterations in left ventricular geometry and function in offspring. Excessive maternal gestational weight gain in the second and third trimesters was associated with interventricular septum thickening, a risk factor for left ventricular hypertrophy in the offspring.

While several intrauterine and maternal risk factors contribute to the EVA trajectories of neonates, there are also non-modifiable factors that predisposes individuals to higher EVA risk, e.g., ethnicity/race, gender/sex, as well as sociocultural and socioeconomic factors. A group of researchers from the Eastern Cape Province in South Africa developed a review of vascular dysfunction and its determinants, with a focus on children of African ancestry (Matjuda et al.). This review is timely, since limited evidence on African normative values exists for either biomarkers measured clinically or in a laboratory and rely on guidelines and reference values from other countries. In children and adolescents, obesity and hypertension remain the main contributors to the development of vascular dysfunction and accelerated vascular aging.

A bidirectional relationship between the large and small arteries (cross-talk) has been described in adults (1), whereby changes in the macrovasculature exacerbates abnormalities in the microvasculature and vice versa. In a study by Breet et al., large artery stiffness was associated with retinal arterial narrowing and venular widening in children, suggestive of cross-talk between the macro- and microvasculature. This study also identified ethnic differences between black and white children, highlighting the need for further investigation into the impact of ethnicity on the macro-microvasculature interactions. In addition to the crosstalk between large and small arteries, as well as the contribution of obesity to EVA, high-risk youth are subjected to reduced diastolic function. Madson et al. illustrated that high arterial stiffness may lead to diastolic dysfunction in youth, and highlighted the importance of prevention and treatment to curb the onset of premature heart failure with preserved ejection fraction in adulthood.

Three studies examined the impact of lifestyle (namely physical activity and/or sedentary behavior) on various markers of vascular aging. In a cross-sectional analysis of 1,324 primary school aged children (7.2 ± 0.4 years), Kochli et al. examined the impact of fitness and obesity on central hemodynamics. This study found that a one-unit increase in body mass index was independently associated with higher central blood pressure and borderline independent higher central pulse pressure, but lower augmentation of the reflected pulse wave. A one-unit increase in shuttle run (stages) was associated with lower central blood pressure, but not after adjustment for confounders, and higher amounts of vigorous activity (min/day) was associated with lower augmentation index. Another study showed that higher amounts of exercise in adolescence is associated with lower cardiovascular risk ~3 years later, however there was no association with carotid IMT (Königstein et al.). Higher volumes of exercise at both baseline and follow up was associated with borderline lower carotid stiffness. Böhm et al., showed in 94 children with a mean age of 12.2 years that the amount of time spent sedentary per day was the strongest predictor of low arterial compliance, but there was no association with endothelial function. Taken together, it is evident from these studies and the existing literature that modifiable lifestyle factors contribute to EVA in childhood.

Type 1 diabetes mellitus (T1DM) is an important cardiovascular risk factor. Although clinically overt cardiovascular complications are extremely rare in children and adolescents with T1DM, many studies have shown that the subclinical features of macro- and microvascular injury are present in diabetic children. Šuláková et al. assessed carotid-femoral pulse wave velocity (cfPWV) in children with T1DM and compared them with an age and sex matched group of healthy, normotensive children. This study showed that children with T1DM, despite similar blood pressure values in ambulatory blood pressure measurements, had higher cfPWV values compared to healthy children. Moreover, the biological age of the arteries estimated by cfPWV in children with T1DM was 5 years higher than in the control group.

A rare disease, tuberous sclerosis, is associated with constitutional activation of the mammalian target of rapamycin (mTOR) pathway. Although experimental studies indicate that mTOR activation causes a disruption of the structure of arteries, there are no studies to assess the structure and elastic properties of arteries in children with tuberous sclerosis. Skrzypczyk et al. showed that children with tuberous sclerosis had higher blood pressure, cfPWV, and carotid intima-media thickness values compared to the age and sex matched group of healthy children. In addition, they found that central blood pressure correlated with renal cysts size.

Given the growing prevalence of cardiovascular risk factors in childhood (2, 3) and the likely impact this will have on EVA, focus should shift to primordial prevention to promote cardiovascular health from early life. For the time being, more work is required to identify the determinants and consequences of EVA in children and adolescents and the most effective and sustainable lifestyle prescription to counteract EVA in early life and to promote longevity.

## Author Contributions

RK, ML, and RC gave substantial contributions to the conception and design of the editorial, drafting the work, revising it critically for important intellectual content, provided approval for publication of the content, and agreed to be accountable for all aspects of the work in ensuring that questions related to the accuracy or integrity of any part of the work are appropriately investigated and resolved. All authors contributed to the article and approved the submitted version.

## Funding

RK is partially funded by the South African Research Chairs Initiative (SARChI) of the Department of Science and Technology and National Research Foundation (NRF) of South Africa (Unique Identification Number: 86895). RC is supported by Australian Heart Foundation Fellowship (102484). None of these funding bodies contributed to the conceptualization, data collection or writing of this paper.

## Author Disclaimer

Any opinion, findings, and conclusions or recommendations expressed in this material are those of the authors, and therefore, the NRF does not accept any liability in this regard.

## Conflict of Interest

The authors declare that the research was conducted in the absence of any commercial or financial relationships that could be construed as a potential conflict of interest.

## Publisher's Note

All claims expressed in this article are solely those of the authors and do not necessarily represent those of their affiliated organizations, or those of the publisher, the editors and the reviewers. Any product that may be evaluated in this article, or claim that may be made by its manufacturer, is not guaranteed or endorsed by the publisher.
